# Impact of Denture Cleaning Method and Overnight Storage Condition on Denture Biofilm Mass and Composition: A Cross-Over Randomized Clinical Trial

**DOI:** 10.1371/journal.pone.0145837

**Published:** 2016-01-05

**Authors:** Joke Duyck, Katleen Vandamme, Stefanie Krausch-Hofmann, Lies Boon, Katrien De Keersmaecker, Eline Jalon, Wim Teughels

**Affiliations:** 1 BIOMAT & Prosthetics section – Department of Oral Health Sciences, KU Leuven & University Hospitals Leuven, Leuven, Belgium; 2 Periodontology section – Department of Oral Health Sciences, KU Leuven & University Hospitals Leuven, Leuven, Belgium; University of North Carolina at Chapel Hill, UNITED STATES

## Abstract

**Background:**

Appropriate oral hygiene is required to maintain oral health in denture wearers. This study aims to compare the role of denture cleaning methods in combination with overnight storage conditions on biofilm mass and composition on acrylic removable dentures.

**Methods:**

In a cross-over randomized controlled trial in 13 older people, 4 conditions with 2 different mechanical cleaning methods and 2 overnight storage conditions were considered: (i) brushing and immersion in water without a cleansing tablet, (ii) brushing and immersion in water with a cleansing tablet, (iii) ultrasonic cleaning and immersion in water without a cleansing tablet, and (iv) ultrasonic cleaning and immersion in water with a cleansing tablet. Each test condition was performed for 5 consecutive days, preceded by a 2-days wash-out period. Biofilm samples were taken at baseline (control) and at the end of each test period from a standardized region. Total and individual levels of selected oral bacteria (n = 20), and of *Candida albicans* were identified using the Polymerase Chain Reaction (PCR) technique. Denture biofilm coverage was scored using an analogue denture plaque score. Paired t-tests and Wilcoxon-signed rank tests were used to compare the test conditions. The level of significance was set at α< 5%.

**Results:**

Overnight denture storage in water with a cleansing tablet significantly reduced the total bacterial count (p<0.01). The difference in total bacterial level between the two mechanical cleaning methods was not statistically significant. No significant effect was observed on the amount of *Candida albicans* nor on the analogue plaque scores.

**Conclusions:**

The use of cleansing tablets during overnight denture storage in addition to mechanical denture cleaning did not affect *Candida albicans* count, but reduced the total bacterial count on acrylic removable dentures compared to overnight storage in water. This effect was more pronounced when combined with ultrasonic cleaning compared to brushing.

**Trial Registration:**

ClinicalTrials.gov NCT02454413

## Introduction

In developed countries, there is a tendency of decreasing edentulism and increased retention of natural teeth until old age [1–4]. Due to an increasing proportion of older people in the population and socio-economic deprivation as persistent risk factor for edentulism [5], the latter remains an oral health issue which is associated with impaired well-being and poor general health [6,7].

Because of their impaired oral function and overall well-being, edentulous persons seek for replacement of their lost teeth. The most common treatment of edentulism in older patients is by means of (implant-supported) removable complete dentures. There is evidence that denture use has indeed a positive association with nutrition, cognitive and physical function, general well-being, and even survival [8–10].Nevertheless, several studies indicate that denture cleanliness and oral hygiene of denture wearers is generally poor [11,12], thereby facilitating the formation and accumulation of an oral biofilm. This biofilm holds a risk for oral infection, discomfort [13–15], and general health problems such as aspiration pneumonia [16,17]. A recent study [18] indicates that the risk for aspiration pneumonia in very old denture wearers (≥ 85 years) even doubles in case they wear their dentures during sleeping as a result of increased microbial load. Denture stomatitis is an oral inflammation related to denture wearing and poor oral hygiene, which occurs in 15 up to 70% of the denture wearers [19–21,13]. Patient-dependent factors, such as specific salivary proteins [22]and host immunity [23] affect the disease.

A variety of denture disinfection methods have been studied for prevention as well as for treatment of denture stomatitis [14,24–30]. A meta-analysis of randomized controlled trials evaluating denture stomatitis treatment strategies was recently performed by Emami et al. [30]. The authors compared the efficacy of antifungal treatment with alternative denture stomatitis treatments such as antiseptic agents and denture disinfection methods. They did not detect statistically significant differences in both clinical and microbiological outcomes between antifungal treatment and different disinfection methods, thereby suggesting that less invasive disinfection methods could be as effective as antifungal therapy to cure denture stomatitis.

The high recurrence rate of the disease [13,31] underlines the importance of prevention of denture (re)contamination and therefore of denture hygiene.

Denture cleaning methods include mechanical and chemical cleaning. Mechanical cleaning implies the removal of plaque using a brush or ultrasonic cleaning. Chemical cleaning products are based on sodium hypochlorite, peroxides, neutral peroxides with enzymes, enzymes or acids. Studies show a positive effect of mechanical and chemical cleaning and of the combination of both on denture cleanliness [24,25,30]. The use of microwave-associated radiation has also been suggested to disinfect dentures [32], but the lack of standardization and inconclusive outcomes of this studies discourage the use of this method as routine denture hygiene measure [26].

Wearing the dentures overnight is also associated with the prevalence of *Candida*-associated stomatitis [14,18,19, 33]. Iinuma et al. [18] indeed observed more tongue and denture plaque, gum inflammation, positive cultures for Candida albicans, and higher levels of circulating interleukin-6 in persons wearing their dentures during sleeping as compared with their counterparts who do not wear their dentures at night.

Despite the recommendation to remove the dentures at night, a significant number of edentulous patients still wears their dentures overnight [34]. Besides, although evidence-based guidelines for denture care and maintenance are available [35], validated guidelines for nocturnal denture storage conditions are missing.

In a previous cross-over randomized controlled trial, we evaluated the effect of different overnight storage conditions, including dry storage, immersion in water, and immersion in water with an alkaline peroxide effervescent tablet on denture biofilm formation and maturation [27]. This study revealed that the use of cleansing tablets significantly reduced denture biofilm mass and pathogenicity compared to dry storage and storage in tap water in case of poor oral hygiene. It remains unclear, however, if the effect of the overnight use of alkaline peroxide effervescent tablets on biofilm mass and composition is influenced in case of preceding mechanical cleaning.

The aim of this study is therefore to investigate the impact of the mechanical cleaning method, combined with overnight storage in water with or without an alkaline peroxide effervescent tablet on denture biofilm mass and composition. It was hypothesized that the use of alkaline peroxide-based effervescent cleansing tablets has no effect on denture biofilm mass and composition in case of preceding mechanical denture cleaning.

Denture brushing is often challenging for older and physically impaired denture wearers [14]. As ultrasonic denture cleaning is reported to be a viable mechanical cleaning alternative [25,28], both denture brushing and ultrasonic denture cleaning are considered as mechanical denture cleaning methods. However, it needs to be clarified if both methods are equally effective. The second hypothesis to be tested in the present study is therefore that there is no difference in denture biofilm mass and composition after denture brushing versus ultrasonic cleaning.

## Materials and Methods

### Study participants

All residents of a Belgian long term care facility (n = 120 residents) for frail older persons were assessed for eligibility to participate in the study. The residents were eligible if they met the following inclusion criteria: being edentulous and wearing complete removable dentures in upper and lower jaw, good oral health (no signs or symptoms of oral disease), good cognitive function to provide written informed consent and to comply with the study requirements. Persons were excluded from the study in case they did not meet the above mentioned inclusion criteria, if they were subject to a current or previous corticosteroid or antimicrobial treatment within 3 months prior to the study, or when they did not wear their dentures all day (e.g. only during the meals).

**Fig 1:**
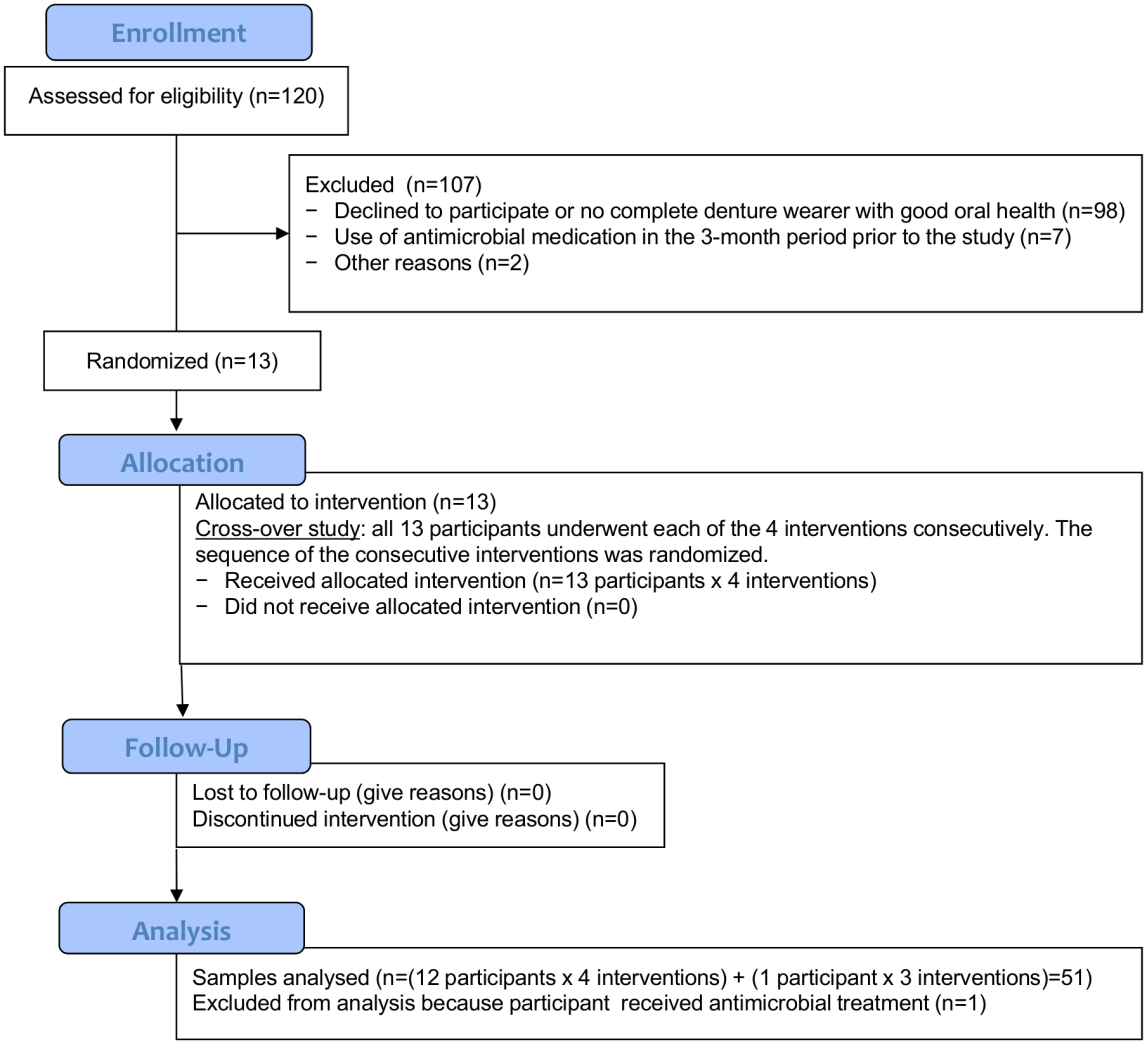
CONSORT 2010 Flow Diagram.

Thirteen patients eventually enrolled in the study and provided written informed consent. Fig 1 presents the CONSORT 2010 Flow Diagram illustrating the participants’ enrollment in the study, allocation, follow-up, and analysis. Due to the time- and work consuming design of the presented study we were explicitly interested in investigating large effects (Cohen's d = 0.8). A power calculation resulted in a minimum number of 11.14 participants that had to be included. The sample size is similar to a previous study [27].

### Study design

The study design is a cross-over randomized controlled clinical study, implying that all study participants were subjected, in a random sequence, to 4 consecutive test conditions with varying mechanical cleaning methods (brushing versus ultrasonic cleaning) and overnight storage conditions (immersion in water with versus without a cleansing tablet):

1. brushing and overnight storage in water without a cleansing tablet (B-T)
2. brushing and overnight storage in water with a cleansing tablet (B+T)
3. ultrasonic cleaning and overnight storage in water without a cleansing tablet (U-T)
4. ultrasonic cleaning and overnight storage in water with a cleansing tablet (U+T)

The sequence of the test conditions was randomized for each participant by LB and KDK. A card was made for each possible sequence and a card was picked blindly for each participant.

Prior to the study, all dentures were decalcified by means of ultrasonic cleaning using a acetic acid-water solution (vinegar), after which these were mechanically cleaned and disinfected using 1% chlorhexidine digluconate gel (Corsodyl gel^®^, GlaxoSmithKline Consumer Healtcare SA, Genval, Belgium) and a prosthesis brush.

Each test condition was performed for 5 consecutive days, preceded by a 2-days wash-out period. During the wash-out period the standard of care of the institution was performed, which consisted of mechanical brushing with water and soap and dry denture overnight storage.

Just prior to and at the end of each test period, the dentures were mechanically cleaned (both brushing with water and soap + additional ultrasonic cleaning in a 0,12% chlorhexidine solution (Perio-Aid^®^, Dentaid, Houten, The Netherlands)) and disinfected by brushing with 1% chlorhexidine digluconate gel (Corsodyl gel^®^, GlaxoSmithKline Consumer Healthcare SA, Genval, Belgium).

Participants and their providers were requested not to clean the dentures themselves during the entire study. A reminder of this was fixed on the participant’s bathroom mirror and denture cleaning materials were removed from the participant’s room. Two researchers (LB, KDK) performed the denture cleaning and applied the appropriate overnight storage condition.

The cleansing tablets used in this study were the *Corega anti-bacteria denture cleanser tablets*^*®*^ (GSK code: 220513, Stafford Miller, Ireland). *Corega anti-bacteria denture cleanser tablets*^*®*^ contain sodium bicarbonate, citric acid, potassium monopersulfate, sodium carbonate peroxide, TAED, sodium benzoate, PEG-180, sodium lauryl sulfoacetate, subtilisin (enzyme), PVP/VA copolymer (film former), aroma’s, and colouring agents (CI 42090, CI 73015, CI 19140). The sodium carbonate peroxide works through an oxygen-liberating process. H₂O₂ oxidizes to release oxygen, which is related to the observed effervescence and is also supposed to exert a mechanical cleansing effect.

The ultrasonic cleaning was performed with the Sonorex Bandelin RK100H device^®^ (Bandelin electronic GmbH & Co. KG, Berlin, Germany) (35kHz) during 15 minutes with tap water at room temperature. After each use, the glass containers holding the dentures were disinfected using 70% ethanol. The mechanical cleaning by means of a denture brush was performed through wet brushing followed by rinsing with tap water.

Mechanical cleaning was completed prior to the denture overnight storage. The researchers LB and KDK performed and controlled the oral hygiene measures and overnight storage. Every evening they cleaned the dentures and stored them for the night according to the protocol. For each patient, there was a cardboard box containing a card with the individual sequence of test conditions and a check-list for the required day-by-day actions. The plastic denture box that contained the denture during the night was disinfected with 70% ethanol after each use.

Microbial control samples (n = 4 per participant) were taken at the start of each test period, after mechanical denture cleaning (both brushing and ultrasonic cleaning) and disinfection to evaluate the effectiveness of the cleaning and disinfection procedures. After each test period (at the end of the 5^th^ day), microbial test samples were taken (n = 4 per participant) and an additional analogue plaque scoring was conducted.

**Fig 2:**
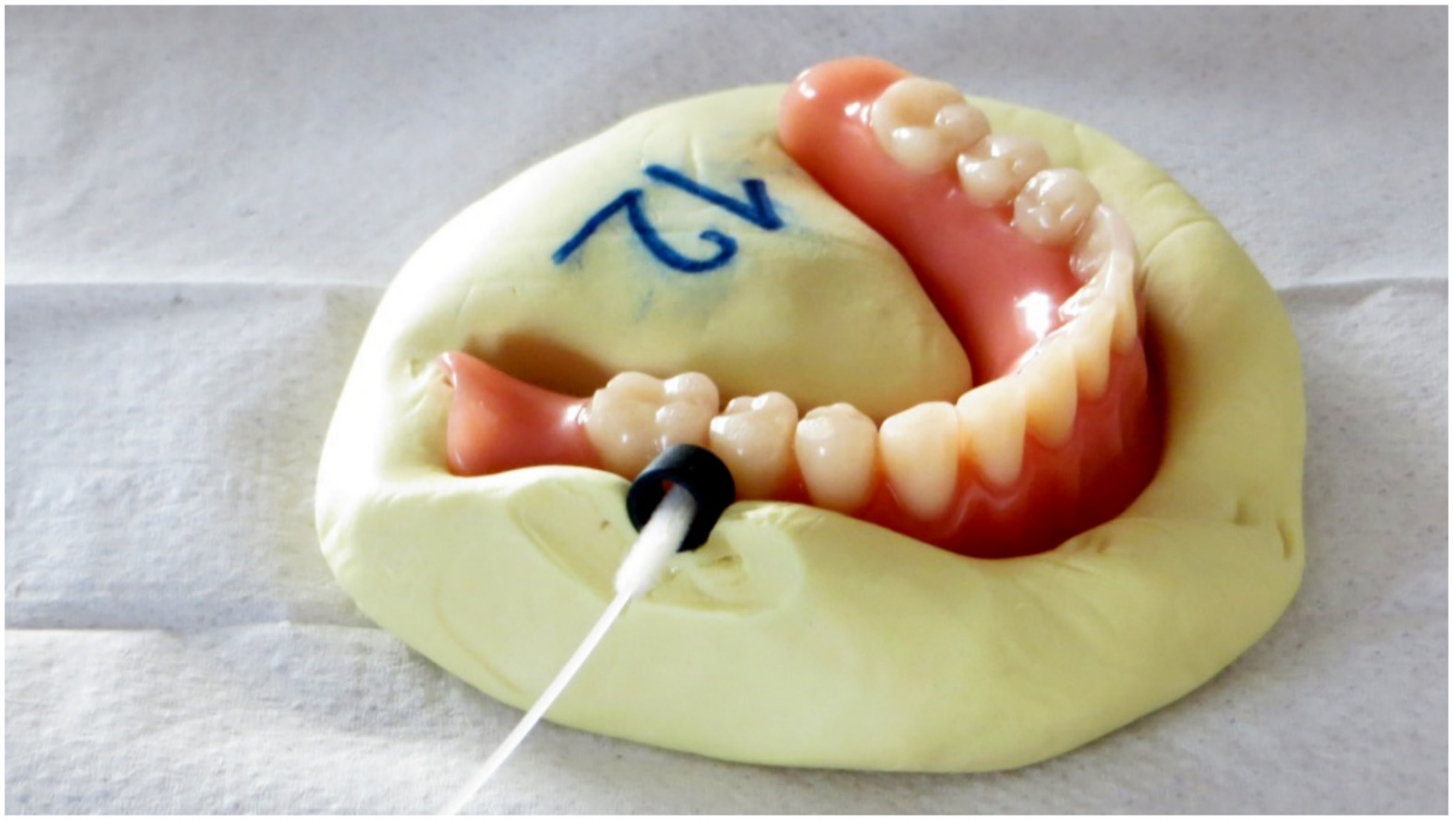
Individualized custom-made mold of the lower denture, made with putty impression material (Optosil^®^, Heraeus Kulzer GmbH, Hanau, Germany) and with placeholder ring *in situ*. The microbial samples were taken within this placeholder ring using sterile swabs (Copan^®^, microRheologics SrL^®^, Brescia, Italy).

The microbial sampling was performed by a researcher (EJ) who had no information on the applied test conditions. The sampling was performed in a 5-mm diameter circular region of interest, situated bucco-distally to the lower second premolars. In order to standardize the position and dimensions of this region and to ensure optimal reproducibility of the microbial sampling, a custom-made mold of each lower prosthesis with placeholder rings was made (Optosil^®^, Heraeus Kulzer GmbH, Hanau, Germany) (Fig 2). The placeholder rings were placed in such a way that the transition between artificial teeth and gums were situated centrally. These molds were fabricated following denture disinfection at the start of the study, and could be re-used throughout the study because of the absence of dimensional changes over time when preserved properly (i.e. dry and in plastic bag). The molds were disinfected using 1% chlorhexidine digluconate gel (Corsodyl gel^®^, GlaxoSmithKline Consumer Healthcare SA, Genval, Belgium) after each microbial sampling and the placeholder rings were ultrasonically cleaned (in Aniosyme dd1^®^, Steralis, Paris, France) and sterilized. The samples were taken using sterile swabs (Copan^®^, microRheologics SrL, Brescia, Italy), removing the biofilm within the contours of the placeholder ring. The swabs were preserved at -18°C. When all samples were collected, they were transferred to Advanced Dental Diagnostics B.V. (Malden, The Netherlands) to perform the qualitative and quantitative PCR analysis for 20 selected oral bacteria (Table 1) and for *Candida albicans*. The classification of Socransky et al. (1998) was used to divide the bacteria into bacterial complexes. The formation of these complexes is based on their association with health or disease severity. The blue, yellow, green and purple complexes designate early colonizers of in early biofilm formation, whereas the red and orange ones are associated with periodontal disease and more matured biofilms [36].

**Table 1:** List of oral bacteria under investigation. The colour codes refer to the classification of Socransky et al. (1998) to divide the bacteria into several bacterial complexes. The formation of these complexes is based on their association with health or disease severity. The blue, yellow, green and purple complexes designate early colonizers of the oral microbiota, whereas the red and orange ones are associated with more matured biofilms and periodontal disease.

Abbreviation		Genus & Species
*Aa*	*Blue complex*	*Aggregatibacter actinomycetemcomitans*
*Pg*	*Red complex*	*Porphyromonas gingivalis*
*Tf*	*Red complex*	*Tannerella forsythensis*
*Td*	*Red complex*	*Treponema denticola*
*Pi*	*Orange complex*	*Prevotella intermedia*
*Fn*	*Orange complex*	*Fusobacterium nucleatum*
*Pm*	*Orange complex*	*Parvimonas micra*
*Pn*	*Orange complex*	*Prevotella nigrescens*
*Cg*	*Orange complex*	*Campylobacter gracilis*
*Cr*	*Orange complex*	*Campylobacter rectus*
*En*	*Orange complex*	*Eubacterium nodatum*
*Ec*	*Green complex*	*Eikenella corrodens*
*Cs*	*Green complex*	*Capnocytophaga species*
*Cc*	*Green complex*	*Campylobacter concisus*
*Smg*	*Yellow complex*	*Streptococcus milleri group*
*Sg*	*Yellow complex*	*Streptococcus gordonii*
*Scg*	*Yellow complex*	*Streptococcus cristatus group*
*Ao*	*Purple complex*	*Actinomyces odontolyticus*
*Av*	*Purple complex*	*Actinomyces viscosus*
*Vp*	*Purple complex*	*Veillonella parvula*

**Fig 3:**
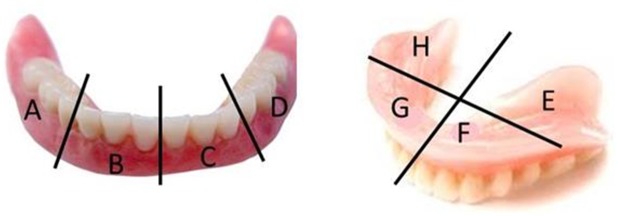
Analogue plaque scoring according to Augsburger & Elahi (1982).

Analogue plaque scoring of the lower dentures was conducted independently by 2 investigators (LB, KDK) using 4% erythrosine disclosing solution according to Augsburger and Elahi [37]. The Augsburger and Elahi method [34] was slightly adapted in order to divide the prostheses in equal areas. The plaque was scored (score 0–4) on 8 zones of the prostheses, 4 at the buccal (ABCD) and 4 at the mucosal side (EFGH) (Fig 3). Scores 0, 1, 2, 3, 4 represent 0%, 1–25%, 26–50%, 51–75%, and 76–100% of the denture zone covered with plaque respectively.

During the study course, neither the study participants nor the lab technicians performing the PCR-analyses were informed about the specific ongoing test condition (blinded set-up).

### Statistical analysis

Analyses were performed using the statistical software SPSS 21. After log-transformation of the data (conversion to log10-values), the number of bacteria count was compared for the following test conditions: 1) overnight storage in water with a cleansing tablet versus without a cleansing tablet (aggregated over the brushing/ultrasound mechanical cleaning), 2) brushing and overnight storage in water without a cleansing tablet versus brushing and overnight storage in water with a cleansing tablet (B-T vs B+T), 3) ultrasound cleaning and overnight storage in water without a cleansing tablet versus ultrasound cleaning and overnight storage in water with a cleansing tablet (U-T vs U+T), 4) brushing versus ultrasound mechanical cleaning (aggregated over the tablet/no tablet condition) (B vs U), 5) brushing and overnight storage in water without a cleansing tablet versus ultrasound cleaning and overnight storage in water without a cleansing tablet (B-T vs U-T), 6) brushing and overnight storage in water with a cleansing tablet versus ultrasound cleaning and overnight storage in water with a cleansing tablet (B+T vs U+T).

These analyses were performed for the total count of bacteria. The Holm-Bonferroni correction method was applied to account for multiple comparisons. To further investigate the effect of the interventions on biofilm composition, analyses were repeated for *Candida albicans* alone as well as for the different complexes of bacteria strains, i.e. the red, orange, green, yellow and purple group according to Socransky et al. [33]. Bacteria complexes with significant differences were further investigated for the individual bacterial strains of the complex. Because of their clinical relevance, individual strains of bacteria of the red and yellow complex were analyzed separately even when not significant at complex-level.

Normality was assessed with Kolmogorov-Smirnov and Shapiro-Wilk statistical tests. Normally distributed data were compared with paired t-tests, and Wilcoxon-signed rank tests were used for nonparametric data. The level of significance was set at α< 5%.

This study was approved by the Institutional Ethics Committee (S54968, University Hospitals Leuven, Belgium) and registered in the Belgian Clinical Trials database (Identifier: B322201316863), as well as in ClinicalTrials.gov (NCT02454413). The study was conducted in August 2013 in Leuven (Belgium) according to the ICH-GCP (International Conference on Harmonization Guidelines on Good Clinical Practice) principles.

## Results

Of the 120 residents of the long term care facility, 98 subjects did not want to participate or did not comply with the inclusion criteria. Most of the persons who did not want to participate did not give a particular reason. The ones who did, mentioned that they did not like the idea of other persons taking care of their dentures or thought it would be difficult for them in one way or another. In addition, one person died, another resident moved away from the long term care facility prior to the start of the study, and seven persons used antimicrobial medication in the 3-month period prior to the study. Therefore 13 eligible subjects agreed to participate in the study. One participant received *nitrofurantoïne* to treat a bladder infection in the last two days of the study. The participant’s data from this last part of the study were therefore not included for analysis. As a consequence, 12 microbial samples were available for the brushing/no tablet (B-T) condition and 13 microbial samples for the other test conditions.

**Fig 4:**
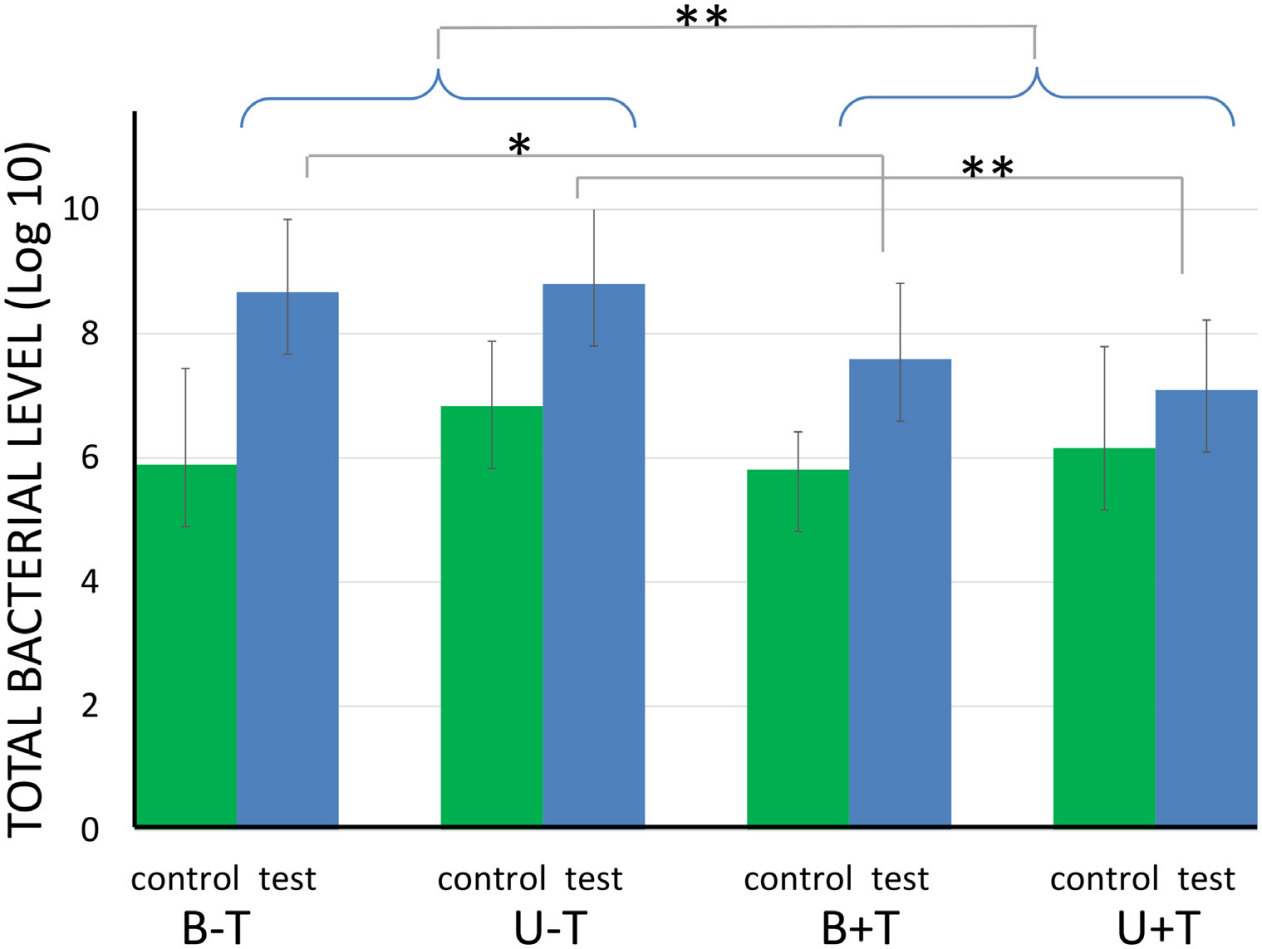
Total bacterial levels for the 4 different test conditions (blue bars) and their respective controls (green bars). B-T: brushing and overnight storage in water without a cleansing tablet; U-T: ultrasonic cleaning and overnight storage in water without a cleansing tablet; B+T: brushing and overnight storage in water with a cleansing tablet; and U+T: ultrasonic cleaning and overnight storage in water with a cleansing tablet. The statistically significant differences are indicated as follows: *p<0.05, **p<0.01 (after Holm-Bonferroni correction).

The mean (standard deviation) bacterial levels for the 4 different test conditions is presented in Table 2. The total bacterial count (log10) was significantly lower for the control (prior to experimental condition) (mean: 6.17, SD: 0.8) compared to the test samples (after experimental condition) (mean: 8, SD: 0.9) (p < 0.001) (Fig 4). No significant differences were observed between the control samples.

**Table 2:** Mean (± standard deviation) Log_10_ CFU/ml for the 4 different test conditions. B-T: brushing and overnight storage in water without a cleansing tablet; U-T: ultrasonic cleaning and overnight storage in water without a cleansing tablet; B+T: brushing and overnight storage in water with a cleansing tablet; and U+T: ultrasonic cleaning and overnight storage in water with a cleansing tablet.

	B-T	B+T	U-T	U+T
**Total count**	8,62 (± 1.13)	7,58 (± 1.22)	8,80 (± 1.22)	7,09 (± 1.13)
**Aa**	0,00 (± 0)	0,00 (± 0)	0,15 (± 0.53)	0,00 (± 0)
**Pg**	0,76 (± 1.00)	1,26 (± 1.07)	1,18 (± 1.03)	1,11 (± 1.10)
**Tf**	2,20 (± 1.27)	2,75 (± 0.66)	1,85 (± 1.62)	2,54 (± 0.54)
**Td**	2,21 (± 0.72)	2,44 (± 0.34)	1,99 (± 0.72)	2,30 (± 0.32)
**Pi**	0,00 (± 0)	0,18 (± 0.66)	0,27 (± 0.98)	0,00 (± 0)
**Fn**	2,97 (± 1.27)	2,63 (± 1.02)	2,99(± 1.46)	2,56 (± 1.01)
**Pm**	0,67 (± 1,66)	0,68 (± 1,66)	0,41 (± 1,48)	0,36 (± 1,28)
**Pn**	0,56 (± 1,37)	0,35 (± 1,27)	0,32 (± 1,14)	0,26 (± 0,91)
**Cg**	0,00 (± 0)	2,97 (± 0.9)	0,67 (± 0)	0,56 (± 0)
**Cr**	1,88 (± 1,1)	1,93 (± 1,38)	1,39 (± 1,73)	2,05 (± 1,26)
**En**	0,00 (± 0)	0,27 (± 0,99)	0,23 (± 0,85)	0,00 (± 0)
**Ec**	3,92 (± 2,36)	2,66 (± 2,58)	3,27 (± 2,75)	1,78 (± 2,37)
**Cs**	2,14 (± 2,36)	1,39 (± 1,62)	2,22 (± 2,2)	0,58 (± 1,4)
**Cc**	3,11 (± 1,94)	2,70 (± 1,92)	3,63 (± 2,32)	1,84 (± 1,94)
**Smg**	6,37 (± 2,12)	5,62 (± 2,07)	6,39 (± 2,22)	5,00 (± 1,85)
**Sg**	1,83 (± 3.03)	1,16 (± 1.92)	1,18 (± 2.43)	2,10 (± 2.23)
**Scg**	2,32 (± 3.01)	2,34 (± 3.10)	3,85 (± 3.20)	1,91 (± 2.54)
**Ao**	4,22 (± 1.82)	3,32 (± 1.26)	4,27 (± 2.14)	2,88 (± 1.25)
**Av**	1,26 (± 2.02)	1,43 (± 1.89)	0,78 (± 1.48)	1,27 (± 2.03)
**Vp**	3,66 (± 2.29)	2,63 (± 1.16)	3,68 (± 2.59)	2,60 (± 0.90)
**Candida**	1,80 (± 2.84)	0,36 (± 1.31)	1,24 (± 2.41)	1,05 (± 2.01)

Overnight denture storage in water with a cleansing tablet significantly reduced the total bacterial count compared to overnight denture storage in water without a cleansing tablet (p<0.01) (Fig 4). This effect was more pronounced in case of ultrasonic cleaning (p<0.01) compared to brushing (p<0.05).

When the bacteria were pooled per complex [33], significantly lower amounts of bacteria of the green and the purple complex were observed in case a cleansing tablet was used (p<0.05). When comparing the test conditions for the individual bacteria, the same held true for Capnocytophaga species (Cs), *Campylobacter concisus* (CC), *Streptococcus milleri group* (Smg), *Actinomyces odontolyticus* (Ao), and *Veillonella parvula* (Vp). This indicates that the use of a cleansing tablet not only affected biofilm mass (total bacterial count), but also the biofilm composition.

The test conditions did not significantly affect the amount of *Candida albicans*.

No significant difference in biofilm mass and composition was found between the 2 mechanical cleaning methods.

**Fig 5:**
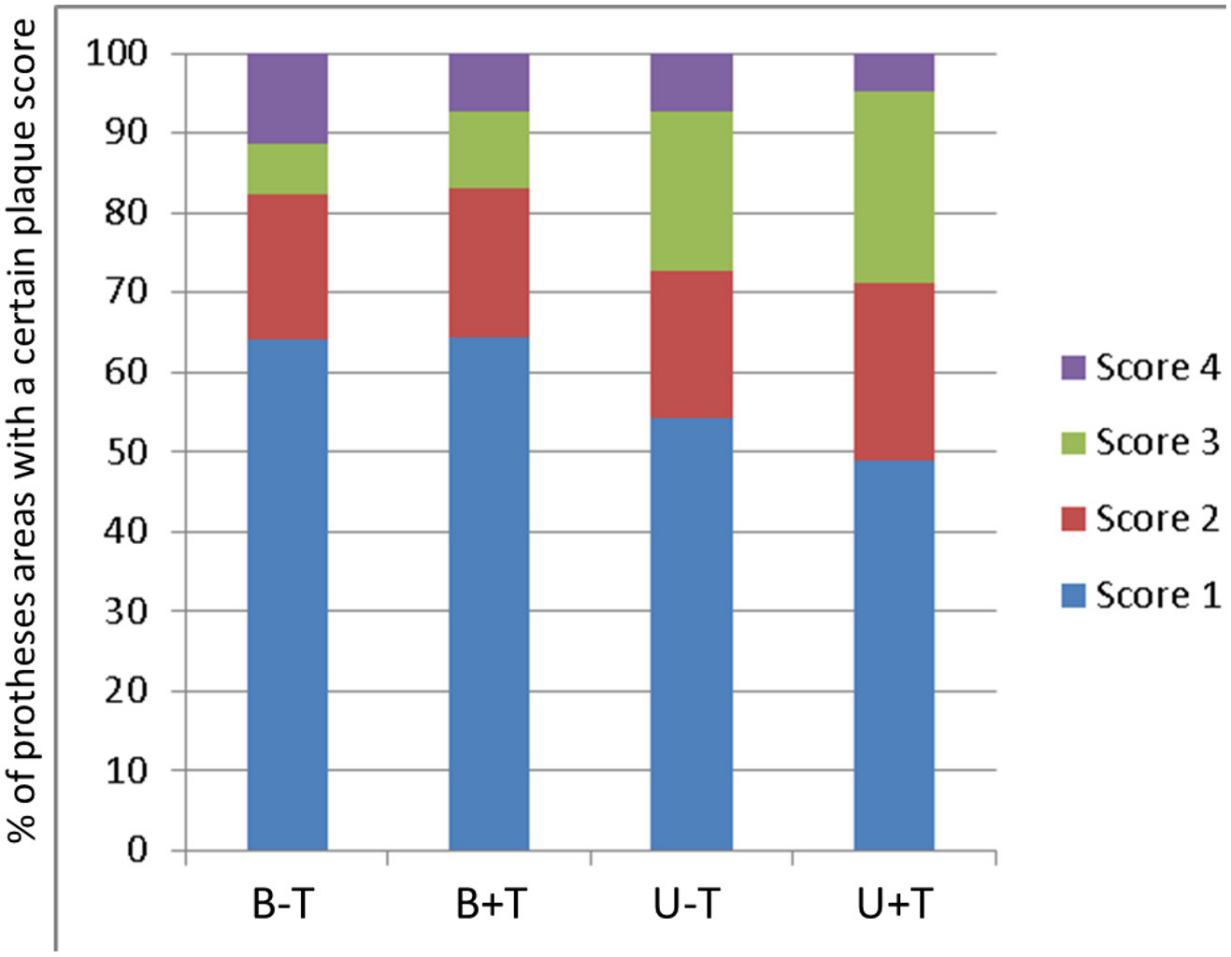
Percentage of the prosthesis areas that received a certain plaque score (score 0: no plaque, score 1: 1–25% covered with plaque, score 2: 26–50% covered with plaque, score 3: 51–75% covered with plaque, score 4: 76–100% covered with plaque).

No significant differences were observed between the analogue plaque scores of both investigators. There were no statistically significant differences in denture plaque score between the different test conditions. Fig 5 presents the percentage of the dentures that received a certain plaque score (calculated on the scores of the 8 separated denture zones). The mean (SD) denture plaque scores for the different test conditions were B-T: 1.65 (0.95), B+T: 1.59 (0.72), U-T: 1.77 (0.70), U+T: 1.85 (0.69).

Significantly (p<0.001) higher plaque scores were observed for the mucosal (average:2.0/SD:1.0) compared to the buccal sides (average:1.4/SD:0.7) of the lower dentures.

## Discussion

Although dentures tend to positively contribute to oral function and general well-being, poor denture hygiene puts the wearer at risk for denture stomatitis, oral malodor, caries and periodontitis on the remaining teeth, and systemic infections associated with oral bacteria [13–15,38]. Appropriate oral hygiene measures are therefore required. Whereas several studies have already been performed on mechanical and chemical denture hygiene techniques [24,25,29], clear guidelines concerning the optimal combination of cleaning method and overnight storage condition are lacking.

In a previous study, we evaluated the effect of different overnight storage methods. Compared to dry storage and immersion in water, immersion of the denture in water with an alkaline peroxide effervescent tablet led to the least total bacterial and *Candida albicans* load in case of poor oral hygiene (no mechanical plaque removal) [27]. Although that study has its value and represents clinical situations with insufficient mechanical cleaning, it did not take into account that usually mechanical denture cleaning is advised prior to overnight denture storage. The question therefore remained whether such cleansing tablets still have an added value in case of thorough mechanical denture cleaning.

The current study was therefore set up to investigate the impact of the combination of a mechanical cleaning method (brushing or ultrasonic cleaning) and an overnight storage condition (using alkaline peroxide effervescent tablets or not) on denture biofilm mass and composition. It was hypothesized that the use of alkaline peroxide-based effervescent cleansing tablets has no effect on denture biofilm formation in case of preceding mechanical denture cleaning.

Significantly more bacteria were found after all test periods compared to at the start of the test period (control). The latter control samples were taken immediately after thorough mechanical cleaning and disinfection of the prostheses in order to provide information on the baseline condition. Although less bacteria were found in these samples, their presence was still considerable. This can be due to the fact that both mechanical cleaning methods do not succeed in removing the denture plaque completely. Porosities in the denture resin can also have contributed to that. In addition, although disinfection using chlorhexidine is effective in killing oral bacteria [36, 39], the PCR-analyses cannot distinguish between dead and living bacteria. It could therefore be that part of the killed bacteria, that are however not physically removed, still contribute to the bacterial counts as measured.

The fact that more bacteria were found in the test compared to the control samples is logical since the test samples were taken at the end of the final day of the test period (24 hours after the last mechanical cleaning session and about 12 hours after the last denture storage condition), whereas the control samples were taken right after denture cleaning and disinfection. Taking the samples at the end of the test period, instead of right after the interventions, provides information about the maximal available biofilm when applying a certain denture hygiene technique and denture overnight storage condition.

Although mechanical cleaning was performed daily, thereby removing all visible plaque, an average denture plaque score of 1.7 on a 0 to 4 scale (SD:0.9) was observed at the end of the test periods. None of the dentures were free from plaque (score 0), 58% of the sites were less than 25% covered with plaque (score 1) and a minor part of the sites (7.7%) were covered more than 75% with plaque (score 4). The different test conditions did not significantly affect the denture plaque scores. This is in contradiction with the findings of Cruz et al. [25] who investigated visual denture plaque with several cleaning methods (overnight denture storage was done in water), including brushing with water 3 times a day (control condition), (1) soaking in effervescent tablets, (2) ultrasonic cleaning and (3) ultrasonic cleaning combined with soaking in a tablet (n = 80). The denture biofilm was scored after 21 days on the internal surfaces of the maxillary complete dentures and revealed less plaque for the test conditions ((1) 37.2%; (2) 35.2%; and (3) 29.1%) compared to the control condition (60.9%). No significant difference was observed between the test conditions. Qualitative investigation of the microbiota was, however, not done in the latter study. Furthermore, the mucosal side of the lower dentures in the current study revealed significantly more plaque compared to the buccal side, which was also reported by other authors [12].

Visual plaque scoring, although used in the majority of the denture plaque studies [24], is a rough method to evaluate biofilm formation. In order to quantify and qualify the denture biofilm more precisely, bacterial samples were taken from a specific site of the denture. PCR analyses of these microbial samples revealed a significant impact of the test conditions on the composition of the biofilm and the number of microorganisms. The way the dentures were cleaned mechanically (brushing compared to ultrasonic cleaning) did not significantly affect the mass and composition of the biofilm. Ultrasonic denture cleaning is not a common denture hygiene technique, probably due to insufficient professional and patient information, although ultrasonic cleaning could be useful in an institutional environment that provides care for physically or cognitively impaired persons [28,29,40]. The current study indicates that the impact of ultrasonic cleaning on biofilm mass and composition is similar to brushing, irrespective whether or not an additional cleansing tablet is used during overnight storage. Our second hypothesis is thereby confirmed.

The use of a cleansing tablet on the other hand significantly reduced the total bacteria count. This effect was more pronounced in case of ultrasonic cleaning (mean difference in total count between U-T and U+T = 1,7) compared to brushing (mean difference in total count between B-T and B+T = 1,1), although B+T did not significantly differ from U+T.

We used the classification of Socransky et al. [36] to divide the bacteria into several bacterial complexes. The formation of these complexes is based on their association with health or disease severity. The blue, yellow, green and purple complexes designate early colonizers in the process of biofilm formation, whereas the red and orange complexes are associated with more matured biofilms and periodontal disease. Bacteria of the purple (*Ao*, *Vp*, *Av*) and the green complex (Ec, Cs, Cc) were significantly reduced when the dentures were stored in water with an effervescent tablet. These bacteria are commensal in the oral biofilm, although they are known as opportunistic pathogens in other parts of the body. Reduction of these bacteria is therefore beneficial, particularly in patients with a decreased immunity [41,42].

In the current study, no significant effect of the test conditions on *Candida albicans* colonization was observed. An early report from Stafford and co-workers [43] evaluated *Candida* colonization after dry overnight storage compared to immersion in water. This study demonstrated that the *Candida albicans* colonization was significantly reduced after 8 hours in dry air. Despite the high risk of bias (e.g. non-standardized sampling sides, no information about denture cleaning), this was for a long time the only study evaluating the effect of overnight denture storage on *Candida albicans* contamination. As no significant effect of the test conditions was observed on *Candida albicans* in the current study, and since the latter is considered a predominant etiological factor for denture stomatitis [13,44,45], it can be questioned whether the added value of storing the denture in water with an effervescent tablet is clinically relevant in case dentures are mechanically well cleaned.

As thorough mechanical cleaning was performed by the researchers in this study, it can be questioned whether this is a best case scenario rather than a clinically representative situation. Indeed, even when patients clean their dentures mechanically, it is likely that there will be more plaque remaining compared to the current study conditions. As studies are available concerning the effect of cleansing tablets on denture hygiene without preceding mechanical cleaning (worst case scenario) [25,27], the clinical reality is likely to be somewhere in the middle.

A comparable study was recently performed by Nishi et al. [29], comparing microorganisms’ survival on complete dentures following ultrasonic cleaning combined with immersion in an effervescent cleansing solution. Fifty full dentures wearers were randomly assigned to 5 groups: 1) immersion in water with an effervescent cleansing tablet (Polident^®^, GlaxoSmithKline Co. Ltd., Tokyo, Japan), 2) brushing with water, 3) ultrasonic cleaning with water, 4) immersion in water with an effervescent cleansing tablet combined with brushing with water, and 5) immersion in water with an effervescent cleansing tablet combined with ultrasonic cleaning. The authors concluded from their study that the use of an effervescent tablet (alone or in combination with mechanical cleaning) was more effective for denture disinfection compared to mechanical cleaning alone, which is in line with the current study. They observed, however, that also the quantity of *Candida albicans* was significantly lower in case of ultrasonic cleaning combined with immersion in water with a cleansing tablet. This finding was not confirmed by the results of the present study. Although the research questions of both studies were similar, the outcome is not entirely equal, which can be attributed to differences in study design. The study of Nishi et al. differs from the current study in that 5 groups of 10 persons were considered in the study [29], whereas the current study performs all test conditions within the same 13 patients (cross-over design), thereby controlling for the patient dependent potential confounding factors (such as saliva quantity and properties, diet, habits). No condition without mechanical cleaning, however, was considered in the current study as this was already tested in our previous study [24]. In the current study, the microbial samples were taken at the start (control) and at the end (test) of a 5-days period in which a specific test condition was applied, whereas the samples of the study by Nishi et al. [29] were taken right before and after application of a certain cleaning moment. Another important difference is the fact that Nishi et al. [29] only measured living microorganisms (through culturing), whereas we identified and counted the microorganisms based on PCR analyses, thereby considering both living and dead microorganisms. The quantification in our study could therefore be an overestimation in case of inefficient mechanical removal of dead microorganisms.

Our study started with a thorough cleaning and disinfection (brushing with 1% digluconate chlorhexidine gel and ultrasonic cleaning in a 0.12% chlorhexidine solution) of the prosthesis after a standardized wash-out period and prior to the test period. This implies that the baseline conditions of the prosthesis for all test period were as standardized as possible and all measures were taken to achieve an optimal disinfection of the prostheses at the start of each test period. This was not done in the study by Nishi et al. [29]. The observed effect of ultrasonic cleaning combined with immersion in water with a cleansing tablet on *Candida albicans* reduction, which was not confirmed by our study, could therefore be the result of the presence of more *Candida albicans* on the prostheses at baseline.

## Conclusion

In conclusion, this study rejects the hypothesis that the use of an effervescent tablet for denture overnight storage has no additional effect on denture plaque mass and composition in case of mechanical denture cleaning. In contrast, a decrease of total bacterial load and of specific bacteria was observed when the dentures were stored in water with an effervescent tablet. No effect, however, could be found on *Candida albicans* colonization, which is considered the main etiological factor for denture stomatitis.

The second hypothesis that there are no differences in denture biofilm mass and composition after denture brushing versus ultrasonic cleaning, however, was confirmed, irrespective of additional overnight denture storage in water with an effervescent cleansing tablet. This indicates that ultrasonic cleaning is an appropriate alternative mechanical cleaning method.

## Supporting Information

S1 Checklist CONSORT 2010 checklist. (PDF)

S1 Protocol The study protocol. (DOC)
