# Supplementary material for: Impact of Denture Cleaning Method and Overnight Storage Condition on Denture Biofilm Mass and Composition: A Cross-Over Randomized Clinical Trial
Source: PLoS One. 2016 Jan 5;11(1):e0145837. doi: 10.1371/journal.pone.0145837 (PMC4701668; doi:10.1371/journal.pone.0145837)
Supplement: S1 Protocol — (DOC) [file pone.0145837.s002.doc]

# ASSESSMENT OF THE IMPACT OF CLEANING METHODS AND OVERNIGHT DENTURE STORAGE ON BIOFILM FORMATION ON REMOVABLE ACRYLIC DENTURES.

**Aim of the study**

The presence of a biofilm – *i.e.* structured microbial communities that are attached to a surface and encased in an exopolymer matrix – on acrylic removable dentures has been associated with serious systemic conditions, especially in the dependent elderly [1]. Oral bacteria have been implicated in bacterial endocarditis [2], aspiration pneumonia [3, 4], chronic obstructive pulmonary disease [5, 6], amongst other diseases [1, 7]. Evidence is available regarding the relationship between proper oral hygiene and overall systemic health.

Plaque on dentures is a complex aggregate containing more than 10^8^ organisms per milligram, and involving more than 600 prokaryote species [8]. The different species collaborate to form a symbiotic biofilm. The biofilms in dentate patients have been studied extensively, but there have been few studies on the biofilm microbiota of complete acrylic dentures [10-18]. It appears that distinct biofilms, with associated pathogenic risks, are present in ‘healthy’ *versus* denture stomatitis affected participants [15, 16, 18]. Not only poor denture cleaning but also inappropriate habits, such as wearing dentures overnight have proved to be associated with the prevalence of *Candida*-associated stomatitis [19, 20]. Therefore, the overnight removal of the acrylic removable dentures is advised in clinical settings. Although evidence-based guidelines for denture care and maintenance are available [21], guidelines for nocturnal storage conditions of dentures are missing. We therefore performed a study[[1]](#endnote-2) (*submitted for publication*) to evaluate the effect of different overnight storage protocols on denture biofilm formation and maturation. The results of this study showed that the use of cleansing tablets (Corega Tabs Anti-bacteria®, GlaxoSmithKline Consumer Healthcare SA, Genval, Belgium) for acrylic removable denture overnight storage reduces denture biofilm mass and pathogenicity compared to dry and water preservation.

As no mechanical denture cleaning was performed in the aforementioned study, the aim of the proposed study is to evaluate the combined effect of mechanical cleaning and overnight storage condition on the microbial composition and *Candida albicans* colonization of denture biofilm formation. This information is required to develop a clinical guideline on acrylic denture cleaning and overnight storage aiming to reduce the risk posed by pathogenic microorganisms, particularly in bedridden and immunocompromised patients.

**Research questions**

- What is the effect of mechanical cleaning on biofilm formation on removable acrylic dentures?
- Is ultrasonic cleaning more efficient compared to denture brushing with respect to prevention of biofilm formation on removable acrylic dentures?
- What is the effect of overnight storage in water with a cleansing tablet on biofilm formation on mechanically cleaned removable acrylic dentures?

**Materials & Methods**

- Participants
- number: 13
- Inclusion criteria:
  - Institutionalised frail elder
  - Fully edentulous in lower and upper jaw
  - Removable denture wearer in lower and upper jaw
  - Good oral health
- Exclusion criteria:
- Current or history of corticosteroid treatment within the 3 months prior to the study
- Current or history of antimicrobial treatment within the 3 months prior to the study
- Inability to provide informed consent
- Inability to comply with the study requirements
- Test conditions

|  | Denture cleaning method |  | Denture overnight storage condition |
| --- | --- | --- | --- |
| 1 | Brushing with water | B+T | Immersion in water with a cleansing tablet |
| 2 | Brushing with water | B | Immersion in water without a cleansing tablet |
| 3 | Ultrasonic cleaning | U+T | Immersion in water with a cleansing tablet |
| 4 | Ultrasonic cleaning | U | Immersion in water without a cleansing tablet |

- Study design
  - Cross-over study with randomized sequence of the 4 test conditions

This implies that all 4 test conditions will be executed within each of the 30 study participants.

- - The sequence of the test conditions for the individual participants will be randomized.
  - Double blind (both the participants as well as the investigators do not know the test conditions)
- All test conditions will be applied for 5 days. This means that all patients will undergo 4 test periods of 5 days.
- Each test period will be preceded by a wash-out period of 2 days in which the standard of care will be applied. This standard of care is denture cleaning through brushing with water and soap and overnight storage in water.
- All dentures will be decalcified using vinegar prior to the start of the study.
- At the start of the study, all dentures will be mechanically cleaned by means of denture brushing and additional ultrasonic cleaning. After mechanical cleaning, the dentures will also be desinfected using a 1% chlorhexidine digluconate gel (Corsodyl gel, GlaxoSmithKline Consumer Healthcare SA, Genval, Belgium).
- After mechanical denture cleaning and desinfection at the start of each test period, control samples (n=4) will be taken to evaluate the effectiveness of the cleaning and desinfection procedures.
- After each test period, test samples (n=4) will be taken
- The participants will be asked not to clean their dentures themselves. 2 care takers involved in the study will do the denture cleaning and will apply the appropriate overnight storage condition.
- Outcome measures:
  - - Clinical health of the denture bearing mucosa

*Method: clinical evaluation*

- - - Microbial analyses

*Method:* ***qualitative and quantitative PCR analyses for 20 selected oral bacteria*** *(Aa, Pg, Tf, Td, Pi, Fn, Pm, Pn, Cg, Cr, En, Ec, Cs, Cc, Sm, Sg, Sc, Ao, Av, Vp) and for* ***Candida Albicans.***

| Abbreviation | Genus & Species |
| --- | --- |
| *Aa* | *Aggregatibacter actinomycetemcomitans* |
| *Av* | *Actinomyces viscosus* |
| *Ao* | *Actinomyces odontolyticus* |
| *Cs* | *Capnocytophaga species* |
| *Cc* | *Campylobacter concisus* |
| *Cg* | *Campylobacter gracilis* |
| *Cr* | *Campylobacter rectus* |
| *Ec* | *Eikenella corrodens* |
| *En* | *Eubacterium nodatum* |
| *Fn* | *Fusobacterium nucleatum* |
| *Pg* | *Porphyromonas gingivalis* |
| *Pi* | *Prevotella intermedia* |
| *Pm* | *Peptostreptococcus micros* |
| *Pn* | *Prevotella nigrescens* |
| *Sc* | *Streptococcus constellatus* |
| *Sg* | *Streptococcus gordonii* |
| *Sm* | *Streptococcus mitis* |
| *Td* | *Treponema denticola* |
| *Tf* | *Tannerella forsythensis* |
| *Vp* | *Veillonella parvula* |

Biofilm sampling will be performed in a 5-mm diameter circular region of interest, situated bucco-distally to the lower second premolars (Figure 1). In order to standardize the position and dimensions of this region and to ensure optimal reproducibility of the microbial sampling, a custom-made mold of each lower prosthesis with PPSU (Polyfenylsulfon) placeholder rings will be made (Optosil®, Heraeus Kulzer GmbH, Hanau, Germany) (Figure 1). The placeholder rings will be placed in such a way that the transition between artificial teeth and gums is situated centrally. These molds will fabricated following denture disinfection at the start of the study, and will be re-used throughout the study because of the absence of dimensional changes over time when preserved properly (*i.e.* dry and in plastic bag). The molds will be disinfected and the rings will be ultrasonically cleaned and sterilized after each microbial sampling.

- - - Denture plaque score

*Method: Denture plaque will be scored after microbial sampling, independently by 2 investigators using 4% erythrosine disclosing solution according to Augsburger and Elahi [22] (score range 0-4)*

- Study protocol

| Time point | Action | Microbial sampling |
| --- | --- | --- |
| Day 0 (morning) | Calculus removal using vinegar  Mechanical denture cleaning ° |  |
| Day 0-2 | Standard of care^i^ |  |
| Day 3 (morning) | Mechanical denture cleaning° and desinfection^ii^ | control sample 1* |
| Day 3-7 | Test period 1 |  |
| Day 7 (evening) | Mechanical denture cleaning° and desinfection^ii^ | test sample 1** |
| Day 8-9 | Standard of care^i^ (wash-out period) |  |
| Day 10 (morning) | Mechanical denture cleaning° and desinfection^ii^ | control sample 2* |
| Day 10-14 | Test period 2 |  |
| Day 14 (evening) | Mechanical denture cleaning° and desinfection^ii^ | test sample 2** |
| Day 15-16 | Standard of care^i^ (wash-out period) |  |
| Day 17 (morning) | Mechanical denture cleaning° and desinfection^ii^ | control 3* |
| Day 17-21 | Test period 3 |  |
| Day 21 (evening) | Mechanical denture cleaning° and desinfection^ii^ | test sample 3** |
| Day 22-23 | Standard of care^i^ (wash-out period) |  |
| Day 24 (morning) | Mechanical denture cleaning° and desinfection^ii^ | control 4* |
| Day 24-28 | Test period 4 |  |
| Day 28 (evening) | Mechanical denture cleaning° and desinfection^ii^  END OF THE STUDY | test sample 4** |

°mechanical denture cleaning: brushing with water and soap + additional ultrasonic cleaning

^i^ standard of care: denture brushing with water and soap and overnight storage in tab water

^ii^desinfection with 1% chlorhexidine digluconate gel (Corsodyl gel, GlaxoSmithKline Consumer Healthcare SA, Genval, Belgium)

*control sampling: after mechanical cleaning and desinfection

**test sampling: before mechanical cleaning desinfection

**References**

1. Coulthwaite L, Verran J. Potential pathogenic aspects of denture plaque. *British Journal of Biomedical Sciences* 2007; **64**:180-89.
2. Que YA, Moreillon P. Infective endocarditis. *Nature Reviews Cardiology* 2011; **8**:322-36.
3. Raghavendran K, Mylotte JM, Scannapieco FA. Nursing homeassociated pneumonia, hospital-acquired pneumonia and ventilatorassociated pneumonia: the contribution of dental biofilms and periodontal inflammation. *Periodontology* 2000 2007; **44**:164-77.
4. El-Solh AA. Association between pneumonia and oral care in nursing home residents. *Lung* 2011; **189**:173-80.
5. Scannapieco FA. Pneumonia in nonambulatory patients: the role of oral bacteria and oral hygiene. *Journal of the American Dental Association* 2006; **137**(10 suppl):21S-25S.
6. Pace CC, McCullough GH. The association between oral microorgansims and aspiration pneumonia in the institutionalized elderly: review and recommendations. *Dysphagia* 2010; **25**:307-22.
7. Senpuku H, Sogame A, Inoshita E, Tsuha Y, Miyazaki H, Hanada N. Systemic diseases in association with microbial species in oral biofilm from elderly requiring care. *Gerontology* 2003; **49**:301-09.
8. Dewhirst FE, Chen T, Izard J, et al. The human oral microbiome. *Journal of Bacteriology* 2010; **192**:5002-17.
9. Mager DL, Ximenez-Fyvie LA, Haffajee AD, Socransky SS. Distribution of selected bacterial species on intraoral surfaces. *Journal of Clinical Periodontology* 2003; **30**:644-54.
10. Theilade E, Budtz-Jørgensen E, Theilade J. Predominant cultivable microflora of plaque on removable dentures in patients with healthy oral mucosa. *Archives of Oral Biology*  1983; **28**:675-80.
11. Moore TC, Smith DE, Kenny GE. Sanitization of dentures by several denture hygiene methods. *Journal of Prosthetic Dentistry*1984; **52**:158-63.
12. Koopmans AS, Kippuw N, de Graaff J. Bacterial involvement in denture-induced stomatitis. *Journal of Dental Research* 1988: **67**: 1246-50.
13. Chan EC, Iugovaz I, Siboo R, et al. Comparison of two popular methods for removal and killing of bacteria from dentures. *Journal of the Canadian Dental Association* 1991; **57**:937-39.
14. Könönen E, Asikainen S, Alaluusua S, et al. Are certain oral pathogens part of normal oral flora in denture-wearing edentulous subjects? *Oral Microbiology and Immunology* 1991; **6**:119-22.
15. Campos MS, Marchini L, Bernardes LA, Paulino LC, Nobrega FG. Biofilm microbial communities of denture stomatitis. *Oral Microbiology and Immunology* 2008; **23**:419-24.
16. Sachdeo A, Haffajee AD, Socransky SS. Biofilms in the edentulous oral cavity. J Prosthodont 2008; **17**:348-56.
17. Pereira-Cenci T, da Silva WJ, Cenci MS, Cury AA. Temporal changes of denture plaque microbiologic composition evaluated in situ. *International Journal of Prosthodontics* 2010; **23**:239-42.
18. Teles FR, Teles RP, Sachdeo A, et al. Comparison of microbial changes in early re-developing biofilms on natural teeth and dentures. *Journal of Periodontology* 2012 Mar 23. [Epub ]
19. Compagnoni MA, Souza RF, Marra J, Pero AC, Barbosa DB. Relationship between Candida and nocturnal denture wear: quantitative study. *Journal of Oral Rehabilitation* 2007; **34**:600-05.
20. Kulak-Ozkan Y, Kazazoglu E, Arikan A. Oral hygiene habits, denture cleanliness, presence of yeasts and stomatitis in elderly people. *Journal of Oral Rehabilitation* 2002; **29**:300-04.
21. Felton D, Cooper L, Duqum I, et al. Evidence-based guidelines for the care and maintenance of complete dentures: a publication of the American College of Prosthodontists. *Journal of Prosthodontics* 2011; **20** Suppl 1:S1-S12.
22. Augsburger RH, Elahi JM. Evaluation of seven proprietary denture cleansers. *Journal of Prosthetic Dentistry* 1982; **47**:356-359.

1. The study was approved by the Institutional Ethics Committee (Catholic University of Leuven, S51463) and registered in the Institutional Clinical Trials database (Identifier: B32220085221), and was conducted according to the ICH-GCP (International Conference on Harmonization Guidelines on Good Clinical Practice) principles. [↑](#endnote-ref-2)
